# Risk Factors of Pancreatic Fistula in Distal Pancreatectomy Patients

**DOI:** 10.1155/2019/4940508

**Published:** 2019-07-17

**Authors:** Amyna Jiwani, Tabish Chawla

**Affiliations:** ^1^Royal Perth Hospital, Perth, Australia; ^2^Aga Khan University Hospital, Karachi, Pakistan

## Abstract

**Introduction:**

Benign and malignant lesions of the pancreas located at the body and tail of the pancreas are managed by the standard procedure of distal pancreatectomy (DP). The mortality associated with this procedure is reported as less than 5% in high-volume centers. The major proportion of morbidity is comprised of pancreatic fistula with a reported incidence of 5% to 60%. The most considered risk factors associated with pancreatic fistula formation are soft pancreatic texture, diameter of the pancreatic duct <3 mm, intraoperative blood loss >1000 ml and surgical techniques. Among all these factors, the modifiable factor is the surgical technique. Several surgical techniques have been developed and modified for closure of the pancreatic remnant in the recent past in order to minimize the risk of pancreatic fistula and other complications. The main objective of the study is to analyze the factors associated with formation of pancreatic fistula after distal pancreatectomy.

**Patients and Methods:**

We performed a single-center retrospective study at Aga Khan University Hospital from January 2004 till December 2015. The perioperative and postoperative data of 131 patients who underwent pancreatic resection were recorded by using ICD 9 coding. 45 patients underwent distal pancreatectomy, out of which 38 were included in the study based on inclusion criteria. Variables were grouped into demographics, indications, operative details, and postoperative course. Statistical analysis software (SPSS) was used for analysis. Quantitative variables were presented as mean with standard deviation or median with interquartile range depending on the distribution of data. Study endpoints for the risk factor analysis were surgical morbidity and development of pancreatic fistula. Univariate logistic regressions were performed associated with study endpoints. *P* value less than 0.05 was considered significant.

**Results:**

Postoperative pancreatic fistula was the most common perioperative morbidity. The significant associated risk factor for pancreatic fistula was multivisceral resection as compared to spleen-preserving distal pancreatectomy (*P* value 0.039). However, the technique of stump closure when opted for suture techniques was seen to be associated with a higher occurrence of postoperative pancreatic fistula. The mortality rate was 2.6%.

**Conclusion:**

Postoperative pancreatic fistula is the most common complication seen after distal pancreatectomy in our series. Multivisceral resection is associated with a high incidence of pancreatic fistula and is a statistical significant predictor of pancreatic fistula.

## 1. Introduction

Benign and malignant lesions of the pancreas located at the body and tail of the procedure are managed by the standard pancreas of distal pancreatectomy (DP). In this procedure, a portion of the pancreas is removed to the left of the pancreas-sparing duodenum and bile ducts. The superior mesenteric vein/portal vein is the landmark for DP, and however, the point of resection of the pancreas depends on the location of the tumor [1, 2].

The mortality associated with this procedure is reported as less than 5% in high-volume centers [3–9], and the morbidity rate remains high ranging from 22% to 50% [10–12]. The major proportion of morbidity is comprised of pancreatic fistula with a reported incidence of 5% to 60% [13, 14]. Pancreatic fistula is defined as a drain output of any measurable volume of fluid with an amylase level >3 times the upper limit of institutional normal serum amylase activity, associated with a clinically relevant development/condition related directly to the postoperative pancreatic fistula [15], and the grades are defined in Table 1. The most considered risk factors associated with pancreatic fistula formation are soft pancreatic texture, diameter of the pancreatic duct <3 mm, intraoperative blood loss >1000 ml and surgical techniques. Among all these factors, it would be interesting to look for a surgical technique which can minimize the risk of postoperative fistula formation, and therefore, for this purpose, this study was conducted. Several surgical techniques have been developed and modified for closure of the pancreatic remnant in the recent past in order to minimize the risk of pancreatic fistula and other complications [1]. A wide range of options are available for the closure of the pancreatic remnant which include the hand-sewn suture technique, the stapled closure technique, or combination of both [11, 16–20], the ultrasonic dissection device [21], pancreaticoenteric anastomosis, application of mesh, seromuscular [22], or gastric mucosa patches [23] or fibrin glue sealants [24, 25]. No study has established any association between pancreatic stump closure and development of pancreatic fistula [26].

The main objective is to analyze the postoperative morbidity and mortality of patients who had undergone distal pancreatectomy for any reason with special attention to the factors contributing to formation of pancreatic fistula. Univariate variables were analyzed to evaluate the impact of these factors in development of pancreatic fistula in these patients.

## 2. Patients and Methods

**Figure 1:**
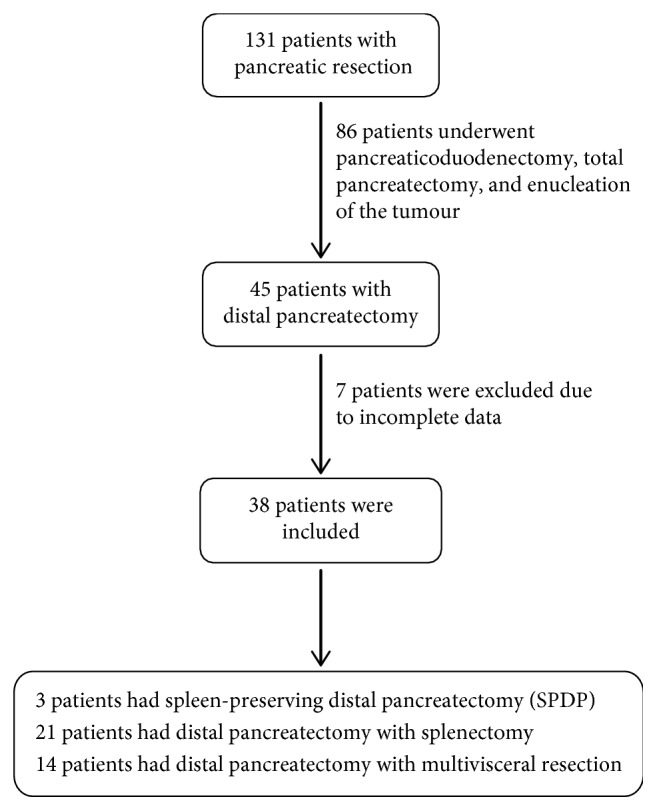
Flow chart of patients included in the study.

Data were retrospectively collected from January 2004 through December 2015 for all the patients admitted for distal pancreatectomy. 131 patients were identified using ICD 9 coding for pancreatic resection, out of which 45 patients had undergone distal pancreatectomy. Thirty-eight patients were included in this study. Ethical approval was obtained from the Ethical Review Committee (ERC) of our institution. Patients who aged 16 years and above and were admitted with pancreatic disease and had undergone laparotomy and distal pancreatectomy were included in this study. Demographics, indications, operative and postoperative course, and morbidity and mortalities were analyzed (Figure 1).

### 2.1. Preoperative Preparations

All the patients had undergone either abdominal computed tomography scan or magnetic resonance cholangiopancreatography (MRCP) or both preoperatively. Prophylactic antibiotics (3^rd^ generation cephalosporin and metronidazole) were given along with DVT prophylaxis preoperatively. Patients were admitted a night before surgery, and preoperative reviews were done. A nasogastric tube was placed after intubation and removed between 1 and 3 days in the postoperative period. Two Jackson–Pratt drains were placed: one at the bed of dissection and another in the pelvis, and these drains were removed between Day 1 and Day 5 depending on the quantity and content. Pain was managed via epidural catheters or patient-controlled analgesia depending on patients' preference and acute pain management service (APMS) of our institution. Pancreatic fistula is defined as the content of drain more than 30 ml per day with high amylase levels (more than three times the serum amylase level), and the grade of pancreatic fistula is defined as per the 2016 update of the International Study Group guideline of pancreatic fistula [15].

### 2.2. Surgical Technique

Upper midline laparotomy is done, and thorough evaluation of the peritoneal cavity is done to look for any other distant disease. The gastrocolic ligament is divided with an energy device (Harmonic or LigaSure) so that the whole pancreas can be visualized. Care should be taken to preserve vessels supplying the stomach. The point of division of the pancreas is decided, and then two stay sutures are placed at the superior and the inferior border. Space is created posterior to the pancreas with blunt dissection. The splenic vein and artery are preserved. Division of the pancreas is done either via sharp dissection or with a stapling device. Distal pancreatectomies are done in a standard fashion. We use either a TCT75 linear staple with an open staple height of 4.0 mm and a closed staple height of 2.0 mm or a contour stapling device. In locally advanced cases, where patients are found to have disease invasion into adjacent organs (stomach, transverse colon, splenic flexure, or spleen), en bloc excision of the tumor with R0 curative intent is undertaken.

### 2.3. Management of Persistent Pancreatic Fistula

Pancreatic fistula is labelled if any of the criteria meets the above-mentioned 2016 update of the International Study Group guideline of pancreatic fistula. Somatostatin is started in the postoperative period for all patients and continued in patients who are being diagnosed with pancreatic fistula, and duration of therapy is dependent on the content and quantity of fluid draining in drain. Ultrasound-guided drain had been placed in patients whose drains were either not working or removed. ERCP is not helpful in such cases, and very rarely, patients need surgical intervention for this condition.

### 2.4. Statistics

Statistical analysis software (IBM SPSS) was used for analysis. Continuous data like age, duration of admission, and duration of surgery were analyzed by mean and standard deviation, and categorical data were by frequencies and percentages. The closure technique and type of surgery were analyzed by Fisher's exact test. Univariate regression analysis was done to identify the association of risk factors with pancreatic fistula. Since only one variable was potentially associated, multivariate analysis was not done.

## 3. Results

A total of 45 patients underwent distal pancreatic resection between January 2004 and December 2015 at the Department of General Surgery at Aga Khan University Hospital, Pakistan. Among 45 patients, 38 patients were included in this study and 7 patients were excluded due to incomplete data.

The mean age of patients who underwent distal pancreatectomy was 41 years with standard deviation of 15 years. 53% of the study population were male, whereas 47% were female. A large proportion of the patients were of ASA II level and ASA III level. 47.3% of the patients had normal BMI, and 49.9% were either overweight or obese. A majority of the patients presented with abdominal pain and weight loss, yet 7.9% of the patients had no symptoms and been diagnosed on incidental findings. The most common indication for distal pancreatectomy was tumor (60.5%). Out of these, only one patient had malignant tumor and rest of them had benign pathology like neuroendocrine tumor (26.3%), pancreatic endocrine tumor (15.3%), and serous cystadenoma (15.3%). Almost all patients underwent CT scan prior to surgery, and only one patient had MRCP (Table 2).

The duration of surgery varied from 149 mins to 277 mins with average mean of 213 mins. Distal pancreatectomy with splenectomy was the most common surgery performed for distal pancreatic pathology. Twenty-four patients had isolated pancreatic pathology who underwent spleen-preserving distal pancreatectomy or distal pancreatectomy with splenectomy and 14 patients had undergone distal pancreatectomy with multivisceral excision of tumor with R0 curative intent who were diagnosed with locally advanced disease involving adjacent organs. The closure technique was variable including hand-sewn, stapled, or both hand-sewn and stapled techniques (Table 2). The most common technique for closure used among surgeons includes both stapled and hand-sewn techniques. Twenty-two patients had hand-sewn with stapled technique used for closure of pancreatic stump, but still hand-sewn and stapled techniques are used separately for closure (18.4% and 23.7%, respectively).

The reported morbidity in our case series is 39.5%, and pancreatic fistula was most commonly seen (21%). Surgical site infection (10.5%), intra-abdominal abscess (10.5%), and septic shock (2.6%) were also identified in patients with some overlapping trend. Only 1 patient required reoperation for intra-abdominal abscess secondary to type C pancreatic fistula, but he recovered and discharged on the 10th postoperative day. Out of 8 patients who were diagnosed with pancreatic fistula, 4 patients had type A, 3 had type B, and 1 had type C pancreatic fistula. In our series, the mortality rate is 2.6% as 1 patient who had underwent multivisceral resection of tumor and had postoperative septic shock due to thoracic duct injury and expired (Table 3).

Univariate analysis was performed to look for association of any risk factor with development of pancreatic fistula. None of the variables like age, BMI, mode of admission, blood transfusion, and ASA level and closure technique showed any significant association with development of pancreatic fistula. Multivisceral resection is statistically significant for postoperative pancreatic fistula formation as compared to spleen-preserving distal pancreatectomy and distal pancreatectomy with splenectomy (Table 4). Because of only one variable showing significance, multivariate analysis was not possible and an independent risk factor could not be calculated in our case series.

## 4. Discussion

The first reported distal pancreatectomy was performed in 1882 by Finney [27] and had been the standard operation since then. It is associated with low morbidity and mortality rate. However, pancreatic stumps in such patients can create problems in the postoperative period. Among all the complications, pancreatic fistula is the most common and troublesome problem which can lead to hemorrhage, abscess formation, sepsis, and septic shock and in worst case, mortality [1, 18, 28]. This can cause a significant impact on healthcare cost burden, especially in developing world with limited resources. Timely identification of this particular complication can limit the catastrophic outcomes. In recent eras, it can be managed conservatively using medications, interventional radiological procedures, and endoscopic procedures, thus significantly decreasing the incidence of mortality [19].

The reported incidence of morbidity in the literature was 22–47% which is similar to the reported morbidity of 39.4% in our study, and the incidence of pancreatic fistula was 21.1% which is similar to that in other studies as well [1, 10, 12, 29]. In 8 patients out of 38 who had pancreatic fistula, half of the patients had type A pancreatic fistula which was managed conservatively without any intervention. 37.5% and 12.5% had type B and type C pancreatic fistula and required some intervention for it. Very few studies have further elaborated the type of fistula formed in their publication. Only Kleef et al. [1] had written its subdivision, and in their study, most of the patients had type B category. In our study, the reported mortality is 2.6% which is coherent with the reported mortality of patients who had undergone distal pancreatectomy.

Multiple systematic reviews had been done in the past to look for any risk factors leading to formation of pancreatic fistula, but none of the studies concluded any definitive answer regarding development of pancreatic fistula because of either limitations of small sample size or nonrandomization of the patients.

The largest series so far published in the literature is by Kleef et al. in 2007 in which he included 302 patients. They studied the association of pancreatic fistula with the technique of pancreatic stump closure. Results showed that 15% of the patients who underwent pancreatic stump closure with the staple technique had pancreatic fistula although 9% in the hand-sewn technique and 8% in the seromuscular patch technique developed pancreatic fistula. They suggested that development of pancreatic fistula is multifactorial and closure technique plays a very crucial part. In this series, closure of pancreatic stump with staple had a high incidence of pancreatic fistula, but it is limited by the retrospective nature of the study [1].

Wellner et al. and Paye et al. presented their data in 2012 and 2014, respectively, and they studied the risk factors associated with formation of postoperative pancreatic fistula. Paye et al. reported that age of 65 years or less, BMI more than 30, and absence of neoadjuvant radiotherapy are associated with pancreatic fistula formation and that multivisceral resection is an independent risk factor for postoperative morbidity, especially pancreatic fistula. This is also established in our results as well [29]. On the contrary, Wellner et al. concluded that high BMI and hand-sewn closure of pancreatic stump are independent risk factors of pancreatic fistula, but our data does not support this finding [12].

This topic had been widely discussed and debated upon, but no conclusive recommendation has been made because of a limited number of patients, nonstandardized techniques, and numerous nonmodifiable factors such as soft pancreas and small duct size. Our series also has some limitations which include different stump closure techniques and different stapling devices (contour or linear staples) used as per the surgeon's preference, and no documentation of pancreatic texture and ductal size was mentioned. We recommend that we need a prospective trial to establish a concrete relation of these factors with formation of postoperative pancreatic fistula.

## 5. Conclusion

Multivisceral resection is associated with postoperative pancreatic fistula formation and increased morbidity. Our data support previous studies that the risks for complications increase with the extensive disease. However, the technique of closure of pancreatic stump may have some effects on occurrence of pancreatic fistula, but it does not reach the value of statistical significance in our study.

## Figures and Tables

**Table 1:** Grade of pancreatic fistula [15].

Grade/type	A (biochemical leak)	B	C
Clinical findings	Well	Often well	Ill appearing/bad
Specific treatment	No	Yes/no	Yes
US/CT scan	Negative	Negative/positive	Positive
Persistent drainage (>3 weeks)	No	Usually yes	Yes
Reoperation	No	No	Yes
Mortality	No	No	Possibly yes
Signs of infection	No	Yes	Yes
Sepsis	No	No	Yes
Reoperation	No	Yes/no	Yes/no

Reproduced from the study of Bassi et al. [15].

**Table 2:** Patients' demographics and operative details.

Variables	Mean with SD (%)
Age	41 ± 15 years
Gender	
Male	53
Female	47
ASA level	
I	2.6
II	65.7
III	26.3
IV	5.2
Body mass index	
Underweight (<18.5)	2.6
Normal (18.5–24.9)	47.3
Overweight (25–29.9)	39.4
Obese (>30)	10.5
Signs and symptoms	
Abdominal pain	57.9
Weight loss	23.7
Nausea and vomiting	18.4
Hypoglycemia	7.9
Incidental findings	7.9
Others^*∗*^	5.2
Duration of admission	11 ± 5 days
Histopathology	
Neuroendocrine tumor (*n* = 10)	26.3
PEN (*n* = 6)	15.8
Serous cystadenoma (*n* = 6)	15.8
Malignant (*n* = 1)	2.6
Others (*n* = 15)^*∗∗*^	39.4
Mode of admission	
Elective	89.5
Emergency	10.5
Imaging used for diagnosis	
CT scan	92.1
MRCP	2.6
Both	5.2
Duration of surgery	213 ± 64 mins
Type of surgery	
Spleen-preserving distal pancreatectomy (*n* = 3)	7.9
Distal pancreatectomy with splenectomy (*n* = 21)	55.3
Distal pancreatectomy with multivisceral excision (*n* = 14)	36.8
Closure	
Hand-sewn (*n* = 7)	18.4
Stapled (*n* = 9)	23.7
Both (*n* = 22)	57.9

^*∗*^Include acute pancreatitis and chronic pancreatitis. ^*∗∗*^Include blunt abdominal trauma with pancreatic laceration in the distal part, penetrating trauma, large bowel tumor invading the distal part of the pancreas, lymphoma, and leiomyosarcoma.

**Table 3:** Postoperative 30-day outcomes.

Outcome	Percentage
Morbidity	39.5
Pancreatic fistula (*n* = 8)	21
Type A (4)	
Type B (3)	
Type C (1)	
Intra-abdominal abscess (*n* = 4)	10.5
Septic shock (*n* = 1)	2.6
SSI (*n* = 4)	10.5
Reoperation (*n* = 1)	2.6
Mortality (*n* = 1)	2.6

**Table 4:** Univariate analysis.

Variables	*n*	Patients with pancreatic fistula	*P* value
Age			
<65 years	34	8	0.560
>65 years	4	0	
BMI			
<25 years	19	3	0.346
>25 years	19	5	
Mode of admission			
Elective	34	7	0.629
Emergency	4	1	
ASA level			
I or II	26	7	0.193
III or IV	12	1	
Pancreatic stump closure			
Hand-sewn	7	3	0.254
Stapled	9	2	
Both	22	2	
Type of surgery			
SPDP	3	0	**0.039**
DP with splenectomy	21	2	
Multivisceral resection	14	6	

## Data Availability

The datasets used to support the findings of this study are available from the corresponding author upon request.
